# Systematically evaluating DOTATATE and FDG as PET immuno-imaging tracers of cardiovascular inflammation

**DOI:** 10.1038/s41598-022-09590-2

**Published:** 2022-04-13

**Authors:** Yohana C. Toner, Adam A. Ghotbi, Sonum Naidu, Ken Sakurai, Mandy M. T. van Leent, Stefan Jordan, Farideh Ordikhani, Letizia Amadori, Alexandros Marios Sofias, Elizabeth L. Fisher, Alexander Maier, Nathaniel Sullivan, Jazz Munitz, Max L. Senders, Christian Mason, Thomas Reiner, Georgios Soultanidis, Jason M. Tarkin, James H. F. Rudd, Chiara Giannarelli, Jordi Ochando, Carlos Pérez-Medina, Andreas Kjaer, Willem J. M. Mulder, Zahi A. Fayad, Claudia Calcagno

**Affiliations:** 1 BioMedical Engineering and Imaging Institute, Icahn School of Medicine at Mount Sinai, 1470 Madison Ave, PO Box: 1234, New York, NY 10029 USA; 2 Diagnostic, Molecular and Interventional Radiology, Icahn School of Medicine at Mount Sinai, New York, NY USA; 3 Department of Internal Medicine and Radboud Center for Infectious Diseases, Radboud University Medical Center, Nijmegen, The Netherlands; 4 Department of Clinical Physiology, Nuclear Medicine and PET and Cluster for Molecular Imaging, Rigshospitalet and University of Copenhagen, Copenhagen, Denmark; 5 Department of Oncological Sciences, Icahn School of Medicine at Mount Sinai, New York, NY USA; 6 Charité – Universitätsmedizin Berlin, Corporate Member of Freie Universität Berlin and Humboldt-Universität Zu Berlin, Institute of Microbiology, Infectious Diseases and Immunology, Berlin, Germany; 7 Department of Genetics and Genomic Sciences, Institute of Genomics and Multiscale Biology, Icahn School of Medicine at Mount Sinai, New York, NY USA; 8 New York University Cardiovascular Research Center, Department of Medicine, Leon H. Charney Division of Cardiology, New York University Grossman School of Medicine, New York University Langone Health, New York, NY USA; 9 Department of Circulation and Medical Imaging, Faculty of Medicine and Health Sciences, Norwegian University of Science and Technology (NTNU), Trondheim, Norway; 10 Department of Cardiology and Angiology I, Faculty of Medicine, Heart Center Freiburg University, University of Freiburg, Freiburg, Germany; 11 Department of Medical Biochemistry, Academic Medical Center, Amsterdam, The Netherlands; 12 Department of Radiology, Memorial Sloan-Kettering Cancer Center, New York, NY USA; 13 Department of Radiology and Chemical Biology Program, Memorial Sloan Kettering Cancer Center, New York, NY USA; 14 Department of Radiology, Weill Cornell Medical College, New York, NY USA; 15 Division of Cardiovascular Medicine, University of Cambridge, Cambridge, UK; 16 Cardiovascular Research Center, Department of Medicine, Icahn School of Medicine at Mount Sinai, New York, NY USA; 17 Transplant Immunology Unit, National Center of Microbiology, Instituto de Salud Carlos III, Madrid, Spain; 18 Centro Nacional de Investigaciones Cardiovasculares (CNIC), Madrid, Spain; 19 Laboratory of Chemical Biology, Department of Biochemical Engineering, Eindhoven University of Technology, Eindhoven, The Netherlands

**Keywords:** Atherosclerosis, Preclinical research, Translational research, Molecular medicine

## Abstract

In recent years, cardiovascular immuno-imaging by positron emission tomography (PET) has undergone tremendous progress in preclinical settings. Clinically, two approved PET tracers hold great potential for inflammation imaging in cardiovascular patients, namely FDG and DOTATATE. While the former is a widely applied metabolic tracer, DOTATATE is a relatively new PET tracer targeting the somatostatin receptor 2 (SST2). In the current study, we performed a detailed, head-to-head comparison of DOTATATE-based radiotracers and [^18^F]F-FDG in mouse and rabbit models of cardiovascular inflammation. For mouse experiments, we labeled DOTATATE with the long-lived isotope [^64^Cu]Cu to enable studying the tracer’s mode of action by complementing in vivo PET/CT experiments with thorough ex vivo immunological analyses. For translational PET/MRI rabbit studies, we employed the more widely clinically used [^68^Ga]Ga-labeled DOTATATE, which was approved by the FDA in 2016. DOTATATE’s pharmacokinetics and timed biodistribution were determined in control and atherosclerotic mice and rabbits by ex vivo gamma counting of blood and organs. Additionally, we performed in vivo PET/CT experiments in mice with atherosclerosis, mice subjected to myocardial infarction and control animals, using both [^64^Cu]Cu-DOTATATE and [^18^F]F-FDG. To evaluate differences in the tracers’ cellular specificity, we performed ensuing ex vivo flow cytometry and gamma counting. In mice subjected to myocardial infarction, in vivo [^64^Cu]Cu-DOTATATE PET showed higher differential uptake between infarcted (SUV_max_ 1.3, IQR, 1.2–1.4, N = 4) and remote myocardium (SUV_max_ 0.7, IQR, 0.5–0.8, N = 4, *p* = 0.0286), and with respect to controls (SUV_max_ 0.6, IQR, 0.5–0.7, N = 4, *p* = 0.0286), than [^18^F]F-FDG PET. In atherosclerotic mice, [^64^Cu]Cu-DOTATATE PET aortic signal, but not [^18^F]F-FDG PET, was higher compared to controls (SUV_max_ 1.1, IQR, 0.9–1.3 and 0.5, IQR, 0.5–0.6, respectively, N = 4, *p* = 0.0286). In both models, [^64^Cu]Cu-DOTATATE demonstrated preferential accumulation in macrophages with respect to other myeloid cells, while [^18^F]F-FDG was taken up by macrophages and other leukocytes. In a translational PET/MRI study in atherosclerotic rabbits, we then compared [^68^Ga]Ga-DOTATATE and [^18^F]F-FDG for the assessment of aortic inflammation, combined with ex vivo radiometric assays and near-infrared imaging of macrophage burden. Rabbit experiments showed significantly higher aortic accumulation of both [^68^Ga]Ga-DOTATATE and [^18^F]F-FDG in atherosclerotic (SUV_max_ 0.415, IQR, 0.338–0.499, N = 32 and 0.446, IQR, 0.387–0.536, N = 27, respectively) compared to control animals (SUV_max_ 0.253, IQR, 0.197–0.285, *p* = 0.0002, N = 10 and 0.349, IQR, 0.299–0.423, *p* = 0.0159, N = 11, respectively). In conclusion, we present a detailed, head-to-head comparison of the novel SST2-specific tracer DOTATATE and the validated metabolic tracer [^18^F]F-FDG for the evaluation of inflammation in small animal models of cardiovascular disease. Our results support further investigations on the use of DOTATATE to assess cardiovascular inflammation as a complementary readout to the widely used [^18^F]F-FDG.

## Introduction

Cardiovascular disease is the principal cause of morbidity and mortality worldwide^1^. In the past two decades, pre-clinical and clinical studies^2–6^ have uncovered inflammation’s critical role in atherosclerotic plaque formation and the onset of cardio- and cerebrovascular events. As cardiovascular inflammation is rapidly developing into a therapeutic target, quantitative positron emission tomography (PET) inflammation imaging of the heart and vasculature is rapidly gaining momentum.

Originally developed as a cancer PET tracer, [^18^F]F-fluorodeoxyglucose ([^18^F]F-FDG) is also the most commonly used inflammation tracer in cardiovascular disease, both in the context of atherosclerosis and cardiac ischemia^7,8^. Nevertheless, several limitations are associated with [^18^F]F-FDG’s use for cardiovascular inflammation imaging. First and foremost, being a glucose analog, [^18^F]F-FDG is taken up by metabolically active cells and is not necessarily specific for inflammatory cells. In fact, in vitro^9^ and recent in vivo atherosclerosis studies^10^ indicate that vascular [^18^F]F-FDG signal might also originate from cells other than plaque macrophages, including non-immune cells. In the context of cardiac imaging, the absence of [^18^F]F-FDG signal is indicative of cardiomyocyte loss after myocardial infarction^11^. Unfortunately, high [^18^F]F-FDG background signal in the healthy heart makes it challenging to visualize inflammation in the coronaries or in the infarct itself^12–14^.

To surpass these limitations, alternative, inflammatory cell-specific PET tracers are being actively investigated for use in cardiovascular disease^12,15–17^. [^68^Ga]Ga- or [^64^Cu]Cu-labeled DOTATATE is a somatostatin receptor type 2 (SST2)-binding, FDA approved, PET radiotracer used to identify and monitor SST2-positive neuroendocrine tumors^18,19^. Since SST2 receptors are also expressed on macrophages^20^, DOTATATE-based tracers have recently gained significant interest for the quantification of inflammation in cardiovascular disease mouse models^21^, in human plaques^20,22^ and, more recently, in myocardial infarction^23,24^. However, the advantages and challenges of using DOTATATE-based radioligands to characterize and quantify cardiovascular inflammation, especially in comparison with the validated and widely used [^18^F]F-FDG, have yet to be elucidated.

Here, we use the combination of in vivo PET imaging and extensive ex vivo radio-immunological assays to study the immunological behavior of [^68^Ga]Ga- and [^64^Cu]Cu-DOTATATE compared to [^18^F]F-FDG, in mouse and rabbit models of cardiovascular inflammation. To facilitate its rigorous evaluation in mice, DOTATATE was labeled with [^64^Cu]Cu. [^64^Cu]Cu’s relatively long radioactive half-life (12.7 h) allows quantitatively studying [^64^Cu]Cu-DOTATATE’s pharmacokinetics, timed biodistribution, quantification of cardiovascular inflammation and cellular specificity over a course of 2 days. Additionally, we performed translational experiments in rabbits on a clinical PET/MRI scanner using [^68^Ga]Ga-DOTATATE (radioactive half-life 68 min), approved by the FDA in 2016, and widely used in humans.

Collectively, our data in several small animal models of cardiovascular disease suggest a valuable complementary role of DOTATATE-based tracers and [^18^F]F-FDG for the assessment of cardiovascular inflammation, supporting the use of DOTATATE in this field.

## Results

### Study design

**Figure 1:**
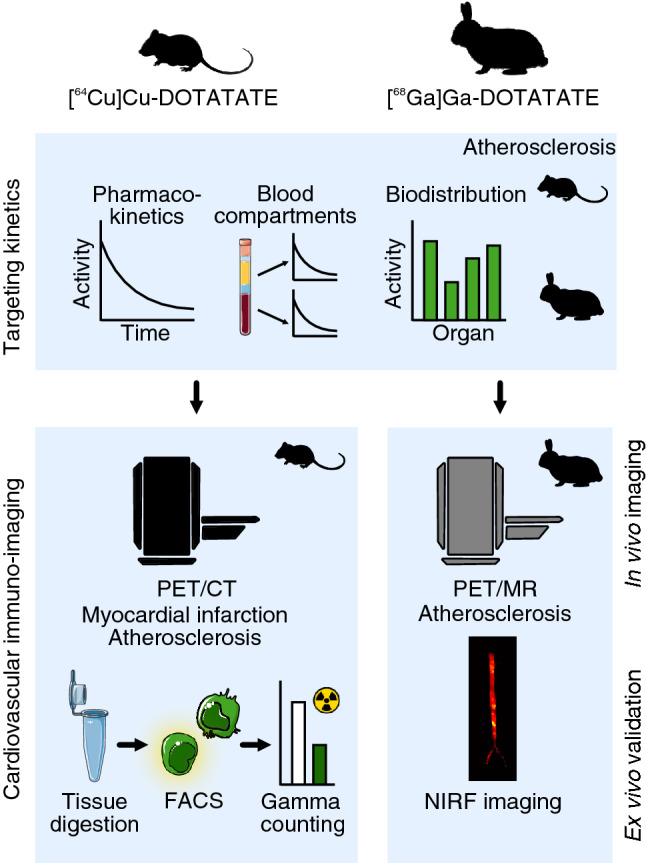
Study design. DOTATATE biodistribution and pharmacokinetics were obtained by ex vivo gamma counting of organs, blood and blood fractions in healthy and atherosclerotic mice and rabbits. In mice, comparison of [^64^Cu]Cu-DOTATATE and [^18^F]F-FDG in models of myocardial infarction and atherosclerosis was performed by in vivo PET/CT, while tracers’ cellular uptake was determined by fluorescence activated cell sorting (FACS) of digested heart and aortic tissue followed by gamma counting. In rabbits, animals were imaged by in vivo PET/MRI using [^68^Ga]Ga-DOTATATE and [^18^F]F-FDG. Results were validated by ex vivo gamma counting and near-infrared fluorescence imaging.

This study’s aim was to thoroughly characterize and compare DOTATATE-based radiotracers and [^18^F]F-FDG for the evaluation of inflammation in mouse and rabbit small animal models of cardiovascular disease (Fig. 1). We designed unique mode of action mouse studies using DOTATATE radiolabeled with the long half-life radioisotope [^64^Cu]Cu (12.7 h). This allows complementing in vivo PET/CT imaging with extensive (and time consuming) ex vivo radiometric and immunological assays. For translational PET/MRI rabbit studies, we used [^68^Ga]Ga-labeled DOTATATE (half-life 68 min), a radiotracer approved by the FDA since 2016, and of high translational relevance due to its use in clinical settings. We investigated DOTATATE’s pharmacokinetics and timed organ biodistribution in mice by ex vivo gamma counting of blood and organs (Supplementary Fig. S1A). In mice with atherosclerosis, mice subjected to myocardial infarction and control animals, we used in vivo PET/CT, together with ex vivo autoradiography and fluorescence activated cell sorting (FACS), to compare the quantification of cardiovascular inflammation and uptake by immune cells between [^64^Cu]Cu-DOTATATE and [^18^F]F-FDG (Supplementary Fig. S1B). Pharmacokinetics and biodistribution were also studied in rabbits (Supplementary Fig. S2A). Subsequently, translational rabbit experiments were performed on a clinical PET/MRI scanner. Atherosclerotic rabbits were imaged with both [^68^Ga]Ga-DOTATATE and [^18^F]F-FDG at different time points after Western diet (WD) initiation and compared to non-atherosclerotic controls. PET/MR data was validated with ex vivo near-infrared imaging of macrophage burden in the aorta (Supplementary Fig. S2B).

### [^64^Cu]Cu-DOTATATE’s in vivo behavior in mice

**Figure 2:**
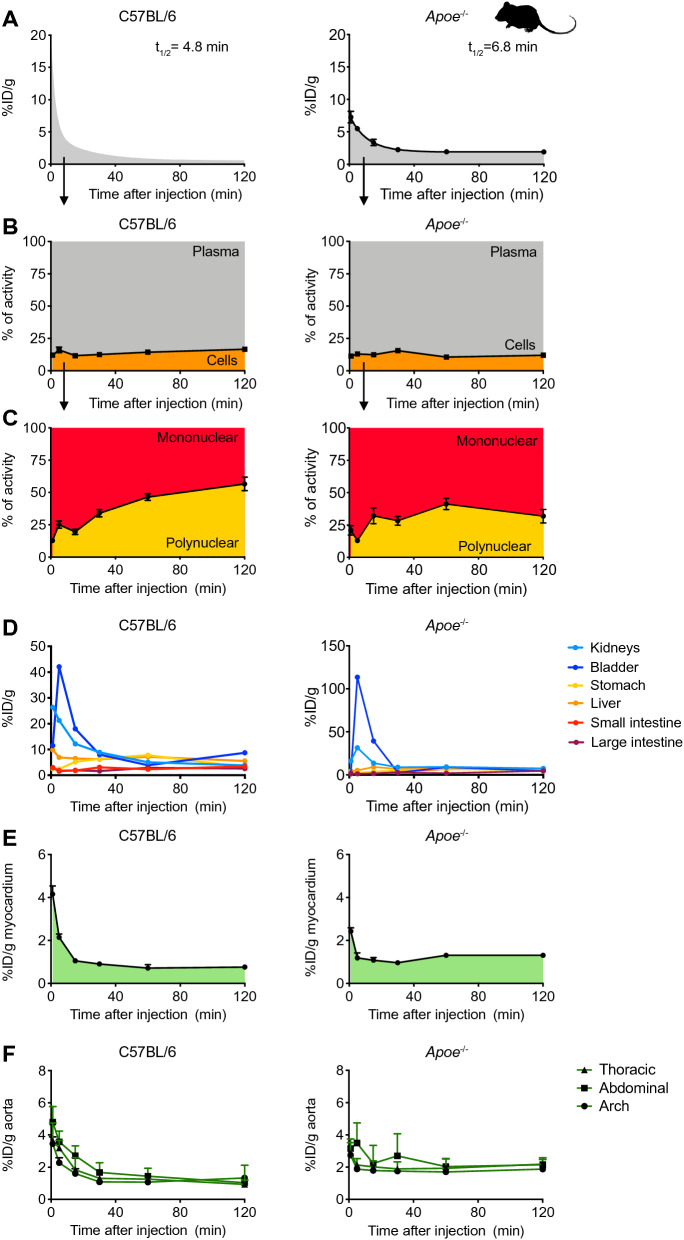
[^64^Cu]Cu-DOTATATE pharmacokinetics and biodistribution in mice. (**A**) Blood time-activity curve for intravenously infused [^64^Cu]Cu-DOTATATE in C57Bl/6 (left) and *Apoe*^−/−^ (right) mice. Data are presented as mean ± standard error of the mean. C57Bl/6 N = 7 and *Apoe*^−/−^ N = 6. (**B**) [^64^Cu]Cu-DOTATATE radioactivity distribution in blood fractions as measured by gamma counting. Graphs show the percentage of activity associated with cells or plasma. Data are presented as mean ± standard error of the mean. N = 5 per group. (**C**) Percentage of activity associated with blood mononuclear or polynuclear cells. Data are presented as mean ± standard error of the mean. N = 5 per group. (**D**) Time-activity curves for [^64^Cu]Cu-DOTATATE in clearance organs. N = 5 per group. (**E**) Gamma counting of [^64^Cu]Cu-DOTATATE activity in the myocardium over time. Data are presented as median (interquartile range). C57Bl/6 N = 5 and *Apoe*^−/−^ N = 4. (**F**) Time-activity curves for [^64^Cu]Cu-DOTATATE in the arch, thoracic and abdominal aorta. Data are presented as median (interquartile range). N = 5 per group. ID: injected dose; t_1/2_: half-life.

In both control and atherosclerotic mice, [^64^Cu]Cu-DOTATATE rapidly cleared from the blood, with a weighted blood half-life of, respectively, 4.8 and 6.8 min (Fig. 2A and Supplementary Table S1, C57Bl/6 N = 7 and *Apoe*^−*/*−^ N = 6). In both groups, separation of blood fractions revealed that most of the tracer was in the plasma, which contained on average 83.4 ± 0.6 and 88.7 ± 0.9% of the radioactivity at 120 min after radiotracer injection, respectively (Fig. 2B, N = 5 per group). Density gradient separation of blood cells indicated that the tracer was taken up by mononuclear cells at early time-points, with progressive increase in polynuclear cells in control and atherosclerotic animals over the 120 min after injection (Fig. 2C, N = 5 per group). In both C57Bl/6 and *Apoe*^−*/*−^ mice, gamma counting of clearance organs substantiated the fast removal of [^64^Cu]Cu-DOTATATE from the bloodstream. Shortly after injection, kidneys, bladder and liver cleared up most of the tracer. [^64^Cu]Cu-DOTATATE exhibited affinity for organs rich in neuroendocrine cells, such as the pancreas, adrenal gland, stomach and intestines (Fig. 2D and Supplementary Fig. S3A, N = 5 per group; Supplementary Tables S2and S3).

To investigate [^64^Cu]Cu-DOTATATE’s suitability for use in cardiovascular disease studies, we evaluated myocardial and aortic uptake over time. In atherosclerotic mice, median heart signal at 120 min was 1.4 (IQR, 1.2–1.4) %ID/g, which was significantly higher compared to 0.7%ID/g in time-matched controls (IQR, 0.7–0.8, *p* = 0.0159, Fig. 2E and Supplementary Fig. S3A; Supplementary Tables S2 and S3, C57Bl/6 N = 5 and *Apoe*^−/−^ N = 4), most likely due to atherosclerosis-driven chronic inflammation. Two hours after injection, signal in the thoracic aorta was also significantly higher in atherosclerotic compared to control mice with medians of 2.1 (IQR, 2–2.4) and 1 (IQR, 0.7–1.1) %ID/g, respectively (*p* = 0.0079, Fig. 2F and Supplementary Fig. S3A; Supplementary Tables S2 and S3, N = 5 per group).

### [^64^Cu]Cu-DOTATATE and [^18^F]F-FDG PET imaging of cardiac inflammation in infarcted mice

**Figure 3:**
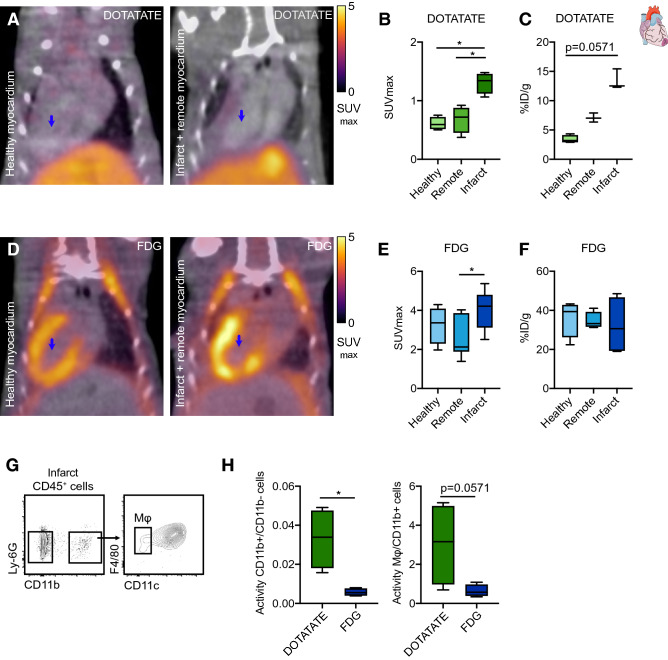
[^64^Cu]Cu-DOTATATE PET imaging of murine myocardial infarction. (**A**) Representative fused [^64^Cu]Cu-DOTATATE PET/CT 3D-rendered images of healthy (left) and LAD-ligated (right) animals. Blue arrows represent region of infarct. (**B**) [^64^Cu]Cu-DOTATATE SUV_max_ (in vivo) and (**C**) %ID/g (ex vivo) in the heart of C57Bl/6 healthy animals (myocardium) and LAD-ligated mice (remote and infarct). N = 4 per group. (**D**) Representative fused [^18^F]F-FDG PET/CT 3D-rendered images of healthy (left) and LAD-ligated (right) animals. Blue arrows represent region of infarct. (**E**) [^18^F]F-FDG SUV_max_ (in vivo) and (**F**) %ID/g (ex vivo) in the heart of C57Bl/6 healthy animals (myocardium) and LAD-ligated mice (remote and infarct). Controls N = 4 and infarcted N = 6. (**G**) Representative flow cytometry plots identifying CD11b^−^, CD11b^+^ and macrophage cell populations in the myocardium. (**H**) Quantification of activity per cell in the infarcted myocardium. Data are expressed as ratio of CD11b^+^/CD11b^−^ (left) and Mφ/CD11b^+^ cell activity (right). N = 4 per group. DOTATATE: [^64^Cu]Cu-DOTATATE; FDG: [^18^F]F-FDG; infarct: infarcted region of the myocardium; ID: injected dose; Mφ: macrophages; Myocardium: healthy C57Bl/6 myocardium; remote: remote myocardium (non-infarcted); SUV_max_: maximum standardized uptake value. CD11b^+^ cells represent CD11b^+^CD11c^hi^, excluding macrophage population. *p < 0.05. Data are presented as median (interquartile range).

Three days after myocardial infarct (MI) induction in C57Bl/6 mice, we performed in vivo PET/CT with either [^64^Cu]Cu-DOTATATE or [^18^F]F-FDG to quantify myocardial inflammation (Supplementary Fig. S1B). [^64^Cu]Cu-DOTATATE PET images showed lower uptake throughout the body, particularly in the muscles (Fig. 3A–C), when compared to [^18^F]F-FDG (Fig. 3D–F). In the infarcted heart, in vivo [^64^Cu]Cu-DOTATATE SUV_max_ was significantly higher (median SUV_max_ 1.3, IQR, 1.2–1.4) compared to both the hearts of control animals (median SUV_max_ 0.6, IQR, 0.5–0.7, *p* = 0.0286), and to the remote (non-infarcted) myocardium (median SUV_max_ 0.7, IQR, 0.5–0.8, *p* = 0.0286, Fig. 3B, N = 4 per group), as confirmed by ex vivo gamma counting and autoradiography (Fig. 3C and Supplementary Fig. S3B, N = 4 per group). In contrast, quantification of [^18^F]F-FDG uptake in control and infarcted hearts showed no significant difference (median SUV_max_ 3.4, IQR 2.6–3.7 and 4.2, IQR 3.3–4.6, respectively, *p* = 0.2571, Fig. 3E, controls N = 4 and infarcted N = 6). Within the heart of infarcted animals, we observed a difference in [^18^F]F-FDG uptake in the infarcted myocardium when compared to remote myocardium (2.1, IQR, 2–3.8, *p* = 0.0411, Fig. 3E, N = 6) as described in the literature^25,26^, but these findings could not be confirmed by ex vivo gamma counting (Fig. 3F, controls N = 4 and infarcted N = 6). By autoradiography, the ratio between the signal in infarcted and remote myocardium was approximately 2 for [^64^Cu]Cu-DOTATATE, but close to 1 for [^18^F]F-FDG (Supplementary Fig. S3B, N = 4 per group), indicating better infarct/remote myocardium delineation using [^64^Cu]Cu-DOTATATE.

We postulate that [^64^Cu]Cu-DOTATATE’s affinity for SST2-expressing cells, particularly macrophages, is responsible for the improved specificity compared to [^18^F]F-FDG. To verify this hypothesis, we harvested the infarcted myocardium and cell-sorted CD11b^+^CD11c^−^F4/80^+^ (macrophages), CD11b^+^ (myeloid cells excluding macrophages) and CD11b^−^ (non-myeloid cells) populations after PET imaging (Fig. 3G, Supplementary Fig. S3C and Supplementary Table S4). Cell suspensions were gamma counted and radiotracer activity was normalized to the number of cells per sample. The ratio of activity in CD11b^+^ (myeloid) versus CD11b^−^ (non-myeloid leukocytes) cells was sixfold higher for [^64^Cu]Cu-DOTATATE compared to [^18^F]F-FDG (*p* = 0.0286, Fig. 3H,  N = 4 per group), indicating higher accumulation of [^64^Cu]Cu-DOTATATE in the myeloid cell fraction. Within CD11b^+^ myeloid cells, [^64^Cu]Cu-DOTATATE was found to accumulate threefold more in macrophages with respect to [^18^F]F-FDG, although these differences did not reach statistical significance (*p* = 0.0571, Fig. 3H, N = 4 per group).

### [^64^Cu]Cu-DOTATATE and [^18^F]F-FDG PET imaging of vascular inflammation in atherosclerotic mice

**Figure 4:**
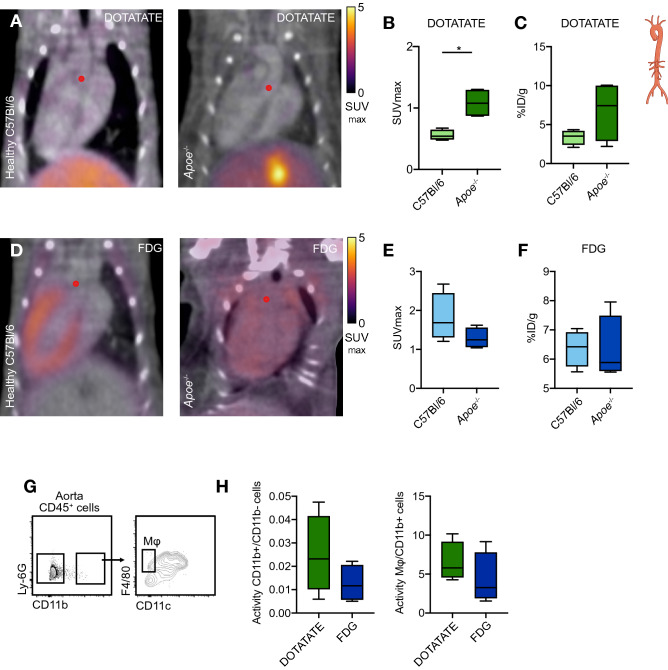
[^64^Cu]Cu-DOTATATE PET imaging of murine atherosclerosis. (**A**) Representative fused PET/CT image of [^64^Cu]Cu-DOTATATE-infused C57Bl/6 (left) and *Apoe*^−/−^ mice after 12 weeks on WD (right). Red dot represents origin of ascending aorta. (**B**) In vivo [^64^Cu]Cu-DOTATATE SUV_max_ of the ascending aorta. N = 4 per group. (**C**) Ex vivo %ID/g of the whole aorta. N = 4 per group. (**D**) Representative fused PET/CT 3D-rendered image of [^18^F]F-FDG-infused *Apoe*^−/−^ mice. Red dot represents origin of ascending aorta. (**E**) [^18^F]F-FDG SUV_max_ of ascending aorta. N = 4 per group. (**F**) %ID/g of the whole aorta. N = 4 per group. (**G**) Representative flow cytometry plots identifying CD11b^+^ and macrophage cell populations in the aorta. (**H**) Quantification of activity per cell in the aorta. Data are expressed as ratio of CD11b^+^/CD11b^−^ (left) and Mφ/CD11b^+^ cell activity (right). N = 4 per group. DOTATATE: [^64^Cu]Cu-DOTATATE; FDG: [^18^F]F-FDG; ID: injected dose; Mφ: macrophages; SUV_max_: maximum standardized uptake value. CD11b^+^ cells represent CD11b^+^CD11c^hi^, excluding macrophage population. *p < 0.05. Data are presented as median (interquartile range).

Encouraged by its macrophage specificity in the heart, we set out to investigate the application of [^64^Cu]Cu-DOTATATE PET imaging in aortic plaques of *Apoe*^−*/*−^ atherosclerotic mice. In vivo PET showed significantly higher [^64^Cu]Cu-DOTATATE SUV_max_ in the ascending aorta of *Apoe*^−*/*−^ mice with median of 1.1 (IQR, 0.9–1.3) compared to healthy (non-atherosclerotic) C57Bl/6 mice where median SUV_max_ was 0.5 (IQR, 0.5–0.6, *p* = 0.0286, Fig. 4A,B, N = 4 per group). This finding was in agreement with the overall trend of ex vivo gamma counting, which, however, did not reach statistical significance, with median %ID/g values of 7.4 (IQR, 3.6–9.9) and 3.5 (IQR, 2.7–4, *p* = 0.2, Fig. 4C, N = 4 per group) in atherosclerotic and control mice, respectively. In atherosclerotic mice, ex vivo autoradiography demonstrated twofold higher signals in atherosclerotic plaques compared to healthy (no plaque) areas of the aorta (Supplementary Fig. S3D, N = 4). [^18^F]F-FDG SUV_max_ in the ascending aorta showed no significant difference between *Apoe*^−*/*−^ mice with median of 1.2 (IQR, 1.1–1.5) and C57Bl/6 control animals with median of 1.7 (IQR, 1.4–2.2, *p* = 0.2, Fig. 4D,E, N = 4 per group), as also observed by ex vivo gamma counting (Fig. 4F, N = 4 per group). In line with our observations in the myocardial infarction model, in atherosclerotic mice there was a non-significant trend for greater uptake of [^64^Cu]Cu-DOTATATE in CD11b^+^ cells over CD11b^−^ cells (twofold higher when compared to [^18^F]F-FDG), and specifically in macrophages (1.5-fold increase when compared to [^18^F]F-FDG, *p* = 0.2, Fig. 4G,H, Supplementary Fig. S3C and Supplementary Table S4, N = 4 per group).

### [^68^Ga]Ga-DOTATATE’s in vivo behavior in rabbits

**Figure 5:**
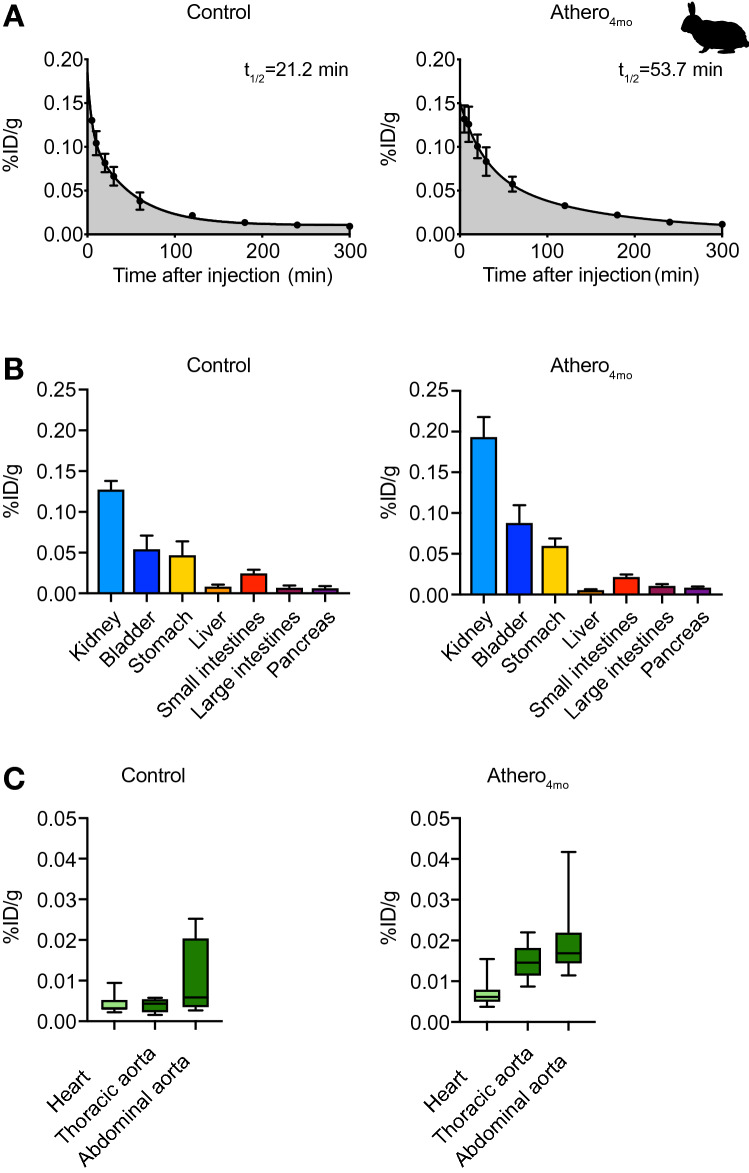
[^68^Ga]Ga-DOTATATE pharmacokinetics and biodistribution in rabbits. (**A**) Blood time-activity curve of [^68^Ga]Ga-DOTATATE-infused control (left) and athero_4mo_ (right) rabbits. Controls N = 2 and athero_4mo_ N = 3. Data are presented as mean ± standard error of the mean. (**B**) Ex vivo quantification of [^68^Ga]Ga-DOTATATE uptake in urinary and digestive organs of rabbits at 200 min after tracer injection, as determined by gamma counting. Data are presented as mean ± standard error of the mean. Controls N = 6 and athero_4mo_ N = 9. (**C**) Ex vivo quantification of [^68^Ga]Ga-DOTATATE uptake in the heart, thoracic and abdominal aorta 200 min after tracer injection, as determined by ex vivo gamma counting. Controls N = 6 and athero_4mo_ N = 9. Data are presented as median (interquartile range). ID: injected dose; t_1/2_: half-life.

In the next stage of our workflow, we evaluated DOTATATE labeled with the short-lived isotope [^68^Ga]Ga in a translational rabbit model of atherosclerosis (aortic endothelium denudation in combination with western diet^27^) on a clinical PET/MR scanner. [^68^Ga]Ga-DOTATATE pharmacokinetics was assessed in atherosclerotic animals 4 months after diet initiation (athero_4mo_) and non-atherosclerotic (control) New Zealand White (NZW) rabbits (Supplementary Fig. S2A). [^68^Ga]Ga-DOTATATE weighted half-life was 53.7 min in atherosclerotic rabbits and 21.2 min in control animals (Fig. 5A and Supplementary Table S1, controls N = 2 and athero_4mo_ N = 3). In line with findings in mice, the majority of [^68^Ga]Ga-DOTATATE was detected in plasma, rather than cells (Supplementary Fig. S4A, controls N = 2 and athero_4mo_ N = 3). [^68^Ga]Ga-DOTATATE behaved similarly to [^64^Cu]Cu-DOTATATE, showing renal clearance and high accumulation in the stomach and other gastrointestinal organs (Fig. 5B and Supplementary Fig. S4B; Supplementary Table S5, controls N = 6 and athero_4mo_ N = 9). In atherosclerotic rabbits [^68^Ga]Ga-DOTATATE median %ID/g in the thoracic and abdominal aorta were, respectively, 0.015 (IQR, 0.011–0.018) and 0.017 (IQR, 0.014–0.022), while, in controls median [^68^Ga]Ga-DOTATATE %ID/g was 0.004 (IQR, 0.002–0.006; *p* = 0.0028) in the thoracic aorta and 0.006 (IQR, 0.003–0.02; *p* = 0.1483) in the abdominal aorta (Fig. 5C and Supplementary Fig. S4B; Supplementary Table S5, controls N = 6 and athero_4mo_ N = 9).

### Translational [^68^Ga]Ga-DOTATATE PET/MR imaging of inflammatory atherosclerosis in rabbits

**Figure 6:**
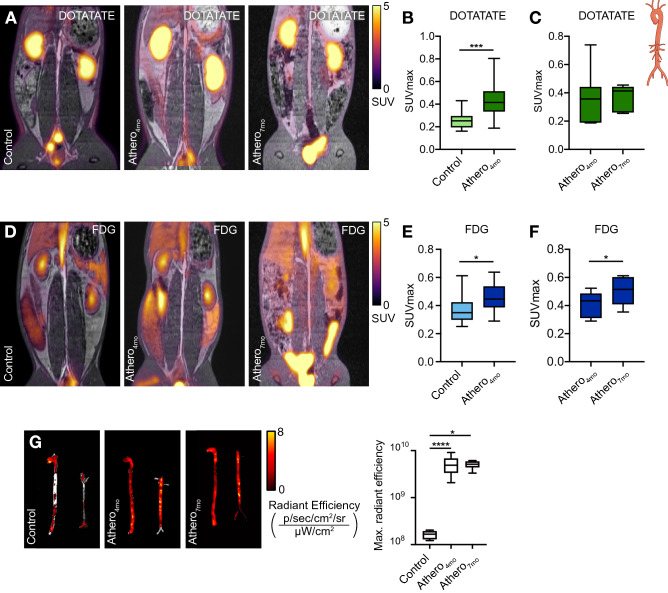
In vivo and ex vivo imaging of atherosclerosis in rabbits. (**A**) Representative fused PET/MR image of [^68^Ga]Ga-DOTATATE-infused control (left), athero_4mo_ (middle) and athero_7mo_ (right) rabbits. (**B**) [^68^Ga]Ga-DOTATATE SUV_max_ of abdominal aorta in control and athero_4mo_ animals. Controls N = 10 and athero_4mo_ N = 32. (**C**) Paired analysis of [^68^Ga]Ga-DOTATATE SUV_max_ of abdominal aorta in athero_4mo_ and athero_7mo_ animals. N = 7 per group. (**D**) Representative fused PET/MR image of [^18^F]F-FDG-infused control (left), athero_4mo_ (middle) and athero_7mo_ (right) rabbits. (**E**) [^18^F]F-FDG SUV_max_ of abdominal aorta in control and athero_4mo_ animals. controls N = 11 and athero_4mo_ N = 27. (**F**) Paired analysis of [^18^F]F-FDG SUV_max_ of abdominal aorta in athero_4mo_ and athero_7mo_ animals. N = 7 per group. (**G**) Near-infrared fluorescence imaging of Cy5.5-HDL in the aorta of rabbits. Controls N = 11, athero_4mo_ N = 15 and athero_7mo_ N = 8. DOTATATE: [^64^Cu]Cu-DOTATATE; FDG: [^18^F]F-FDG; SUV_max_: maximum standardized uptake value. *p < 0.05, ***p < 0.001, ****p < 0.0001. Data are presented as median (interquartile range).

Encouraged by the mouse data, we performed an extensive PET imaging study to quantify vascular inflammation by [^68^Ga]Ga-DOTATATE’s and [^18^F]F-FDG in an atherosclerosis rabbit model. Atherosclerotic rabbits 4 and 7 months after Western diet initiation (athero_4mo_ and athero_7mo_, respectively) and healthy controls were imaged with both [^68^Ga]Ga-DOTATATE and [^18^F]F-FDG on separate days (Supplementary Fig. S2B). Based on pharmacokinetic analyses and dynamic imaging experiments (Supplementary Fig. S4C, N = 3 per group), we allowed [^68^Ga]Ga-DOTATATE to circulate for 120 min and [^18^F]F-FDG for 180 min, as previously validated^28^, before PET imaging. Using [^68^Ga]Ga-DOTATATE (Fig. 6A), we found a 1.6-fold higher SUV_max_ in atherosclerotic aortas of athero_4mo_ animals as compared to controls, with medians of 0.415 (IQR, 0.338–0.499) and 0.253 (IQR, 0.197–0.285), respectively (*p* = 0.0002) (Fig. 6B, controls N = 10 and athero_4mo_ N = 32, excluded rabbits N = 5). However, based on [^68^Ga]Ga-DOTATATE PET, we did not detect a further increase in tracer accumulation in rabbits kept on Western Diet for 7 months (athero_7mo_, *p* > 0.9999, Fig. 6C, N = 7, excluded rabbits N = 2). Similar to previous studies^29^, we found 1.2-fold higher [^18^F]F-FDG SUV_max_ in the abdominal aorta of athero_4mo_ rabbits, with median 0.446 (IQR, 0.387–0.536), as compared to controls, with median 0.349 (IQR, 0.299–0.423, *p* = 0.0159, Fig. 6D,E, controls N = 11 and athero_4mo_ N = 27, excluded rabbits N = 9). [^18^F]F-FDG SUV_max_ also increased to a median of 0.515 (IQR, 0.409–0.602) over 3 additional months of Western diet (athero_7mo_, *p* = 0.0312, Fig. 6F, N = 7, excluded rabbits N = 2). Analysis of ex vivo near-infrared fluorescence imaging of Cy5.5-labeled HDL in the aorta (a validated marker of macrophage burden^30^) was in line with [^68^Ga]Ga-DOTATATE results: while a significant difference was found in maximum radiant efficiency median values between control 1.67 × 10^8^ (IQR, 1.29 × 10^8^–1.94 × 10^8^) and atherosclerotic athero_4mo_ animals 4.89 × 10^9^ (IQR, 3.36 × 10^9^–6.79 × 10^9^, *p* < 0.0001), no increase in fluorescent HDL signal was observed when comparing athero_4mo_ and athero_7mo_, suggesting no increase in macrophage burden over time (*p* = 0.5584, Fig. 6G, controls N = 11, athero_4mo_ N = 15 and athero_7mo_ N = 8). A weak, but significant (r = 0.3522; *p* = 0.0141), correlation was found between [^18^F]F-FDG and [^68^Ga]Ga-DOTATATE aortic SUV_max_, reflecting the partially overlapping cellular specificity of the two tracers (Supplementary Fig. S4D).

## Discussion

The use of SST2-specific PET radiotracers, and predominantly DOTATATE-based ligands, is rapidly gaining momentum for cardiovascular inflammation imaging^8,26,31^. In pre-clinical settings, visible uptake of SST2-specific PET radiotracers^15,21^ including, but not limited to, DOTATATE has been previously demonstrated in mice with atherosclerosis. Yet, in selected studies, in vivo vascular imaging findings were confounded by concomitant uptake in nearby organs. Following successful retrospective analyses in cancer patients^32,33^, DOTATATE vascular uptake has been shown in the vasculature of patients with coronary and carotid atherosclerosis^20,34^, although other studies have question DOTATATE’s ability to discriminate symptomatic lesions in humans^35^. Studies in patients with myocardial infarction^23^ and a case report on cerebral stroke^36^ have demonstrated significant uptake of DOTATATE in the infarcted, with respect to non-infarcted, tissue.

However, the strengths and weaknesses of DOTATATE, especially with respect to the validated metabolic tracer [^18^F]F-FDG, in evaluating cardiovascular inflammation still need to be fully elucidated. In this study, we sought to thoroughly characterize the in vivo immune behavior of the SST2-specific [^68^Ga]Ga- and [^64^Cu]Cu-DOTATATE in comparison with the widely used metabolic tracer [^18^F]F-FDG in experimental models of cardiovascular disease. Previous studies suggest that [^64^Cu]Cu-DOTATATE and [^68^Ga]Ga-DOTATATE have similar imaging performance for detection of neuroendocrine tumors. In the literature, dosimetric data indicates that differences in DOTATATE’s tumor effective dose for both tracers is negligeable^37,38^ and further image analysis in animal models showed similar results^39^.

In mechanistic mouse experiments, we labeled DOTATATE with [^64^Cu]Cu. The longer physical half-life of this radioisotope allowed complementing in vivo PET imaging with extensive ex vivo assays, including flow cytometry, gamma counting, and autoradiography, to investigate the tracer pharmacokinetics, timed organ biodistribution, cellular specificity and ability to report on cardiovascular inflammation. In translational PET/MRI rabbit studies, we instead used in vivo PET imaging with [^68^Ga]Ga-DOTATATE to assess aortic inflammation, in comparison with [^18^F]F-FDG.

Our pharmacokinetic studies in mice confirmed rapid clearance of [^64^Cu]Cu-DOTATATE from the bloodstream (similar to [^18^F]F-FDG, which has a 5.1 min half-life)^40^. This feature is important when performing in vivo imaging and especially in the vasculature, whose signal can be easily contaminated by the blood pool because of partial volume errors. Timed biodistribution analysis in the mouse confirmed fast accumulation in the kidneys and gastrointestinal organs^41^, and, as expected, targeting of SST2-expressing organs, such as pancreas and adrenal gland. We further used the combination of in vivo PET/CT imaging and ex vivo radiometric and immunological assays to investigate [^64^Cu]Cu-DOTATATE as an inflammation tracer in mice with atherosclerosis and mice subjected to myocardial infarction. For the myocardial infarction model, mice were imaged 3 days after LAD ligation surgery. This timeline was chosen to capture the peak of SST2-expressing inflammatory macrophage infiltration in the infarcted myocardium^26^, since SST2 is upregulated in inflammatory macrophages/LPS-stimulated macrophages, while expression in other leukocytes is negligible^20,42,43^. A previous preclinical study^24^ that investigated the use of [^68^Ga]Ga-DOTATATE in mouse models of cardiac ischemia did not show significant radiotracer accumulation in the infarct. While we also generally observed low myocardial signal, our data suggests improved infarct delineation from healthy myocardium with [^64^Cu]Cu-DOTATATE in comparison with [^18^F]F-FDG, as confirmed by in vivo SUVmax measurements. The difference between our findings and previous studies employing [^68^Ga]Ga-DOTATATE may be due to the lower positron range of [^64^Cu]Cu (1 mm) with respect to [^68^Ga]Ga (4 mm), which intrinsically lowers the occurrence of partial volume artifacts from the blood stream, while offering intrinsic better signal-to-noise and spatial resolution^19,22,44^. Our results are also in agreement with findings from a recent clinical study^23^,where focal, infarct-related [^68^Ga]Ga-DOTATATE signal in patients with MI was well-visualized thanks to the tracer very low physiological background tracer uptake, and was significantly higher than in the remote myocardium. In line with several clinical studies, as well as previous ex vivo autoradiography and in vivo analysis in *Apoe*^−*/*−^ mice using [^68^Ga]Ga-DOTATATE^20–22,33,34,41,45^, we confirmed accumulation of [^64^Cu]Cu-DOTATATE in mouse aortic plaques. Pre-clinical studies applying different SST2-specific PET radiotracers, such as the antagonist [^111^In]In-DOTA-JR11, in atherosclerotic *Apoe*^−*/*−^ mice have also demonstrated visible tracer accumulation in plaques. However, the interpretation of in vivo imaging findings was hampered by thymus uptake^15^, making it challenging to use these different tracers in pre-clinical research settings. Our atherosclerosis mouse model also showed that [^64^Cu]Cu-DOTATATE uptake was higher in the ascending aorta of atherosclerotic versus control animals, while no differences were detected for [^18^F]F-FDG. While other studies in mice showed higher [^18^F]F-FDG accumulation in atherosclerotic versus control animals^46,47^, a recent report indicated that the specific anesthesia regimen used, and periaortic fat uptake may significantly affect [^18^F]F-FDG plaque signal^48^. [^64^Cu]Cu-DOTATATE blood half-life was longer in atherosclerotic mice (and rabbits) compared to controls, a factor that may potentially confound vessel wall readings because of higher blood background signal. We hypothesize that this phenomenon may be attributed to lower renal clearance (due to impaired kidney function) in diseased animals. However, ex vivo gamma counting validated the higher aortic tracer accumulation in atherosclerotic versus healthy animals, thereby mitigating these concerns.

Unlike a recently published in vitro analysis that employed macrophages differentiated from an immortalized THP-1 cell line^49^, flow cytometry in both mouse models from our study confirmed higher affinity of DOTATATE for myeloid CD11b^+^ cells, with respect to [^18^F]F-FDG, particularly for the macrophage fraction. This discrepancy might be attributed to methodological differences between the two studies, such as our studies being conducted in vivo, in mouse models, as opposed to in vitro, in cells derived from human blood, as well as the different cell isolation protocols. Our results are in line with a recent clinical study that confirmed high expression of SST2 in human M1 inflammatory macrophages, in comparison with other myeloid and immune cells. However, in the same study, glucose transporters 1 and 3 (GLUT1 and GLUT3) were found to be highly expressed by all immune cells, corroborating the lower cellular specificity of [^18^F]F-FDG^20^.

In addition to the extensive mechanistic mouse work, we evaluated [^68^Ga]Ga-DOTATATE in a rabbit model of atherosclerosis. Ex vivo quantification of aortic HDL accumulation (a marker of macrophage burden) by near-infrared fluorescence imaging was in line with the in vivo [^68^Ga]Ga-DOTATATE readout, indicating no increased macrophage burden during atherosclerosis progression. These findings strengthen the notion that the two tracers report on inter-related but intrinsically different processes.

To summarize, in this study we present a detailed, head-to-head comparison of the novel SST2-specific tracer DOTATATE and the validated metabolic tracer [^18^F]F-FDG for the immune evaluation of inflammation in small animal models of cardiovascular disease. Our encouraging results support DOTATATE’s use to assess cardiovascular inflammation, as a complementary readout to the widely used [^18^F]F-FDG PET.

## Methods

### Radiolabeling of DOTA-(Tyr^3^)-Octreotate with Copper-64 [^64^Cu]Cu

DOTA-(Tyr^3^)-Octreotate (DOTATATE) acetate salt was purchased from Bachem (Bubendorf, Switzerland). [^64^Cu]Cu was produced using a CS15 cyclotron located at Washington University by the [^64^Ni]Ni(p,n)[^64^Cu]Cu nuclear reaction. Activity measurements were made using a Capintec CRC-15R Dose Calibrator (Capintec, Florham Park, NJ). [^64^Cu]Cu was chelated to DOTATATE by adjusting the pH of a [^64^Cu]Cu solution to 5, using a buffer containing 0.1 M NH_4_OAc. Subsequently, 20 µg of DOTATATE were added to the solution and the reaction mixture was heated at 80 °C for approximately 45 min. The labeling reaction yielded [^64^Cu]Cu-DOTATATE with a radiochemical purity of > 98%, as determined by RadioHPLC, and a representative specific activity of 10 MBq/nmol. [^68^Ga]Ga-DOTATATE was purchased from Advanced Accelerator Applications (Millburn, NJ), with a representative specific activity of 1.01 MBq/nmol.

### High-performance liquid chromatography (HPLC) and radio-HPLC

High-performance liquid chromatography (HPLC) was performed on a Shimadzu (Kyoto, Japan) HPLC system equipped with two LC-10AT pumps and an SPD-M10AVP photodiode array detector. Radio-HPLC was performed using a Lablogic (Tampa, FL) Scan-RAM Radio-TLC/HPLC detector. Reverse phase chromatography was performed using a Waters Atlantis T3 column, 100, 5 µm, 4.6 mm × 250 mm (Waters, Milford, MA) with an acetonitrile to water gradient from 5 to 95% acetonitrile over 20 min at a flow rate of 1.0 mL min^−1^.

### Animal experiments

All animal experiments were performed in accordance with protocols approved by the Institutional Animal Care and Use Committee at the Icahn School of Medicine at Mount Sinai and followed National Institutes of Health guidelines for animal welfare. This study is reported in accordance with ARRIVE guidelines.

### Mouse model

Animals were housed under a constant room temperature at 25 ± 2 °C and 50 ± 5% humidity with a 12-h daylight period and 12-h darkness period, with free access to water. For the mouse atherosclerosis model, 8-weeks old female *Apoe*^−/−^ mice (N = 51) were purchased from Jackson Laboratories (Bar Harbor, ME) and, after a 48 h acclimatization period, were fed a Western Diet (42% Kcal from fat TD88137, Envigo, Huntingdon, UK) for 12 weeks. 16–20 weeks old female C57Bl/6 controls (N = 67) were kept on regular chow diet. The myocardial infarction group consisted 16–20 weeks old female C57Bl/6 mice (N = 18) purchased from Jackson Laboratories and subjected to ligation of the left anterior descending artery, as previously described^50^.

### Rabbit model

For the rabbit studies, 3 months old male New Zealand White (NZW) rabbits (N = 47) were purchased from Charles River Laboratories (Wilmington, MA). Randomly assigned control rabbits (N = 11) were kept on chow diet and imaged with PET/MR. Among these some animals were euthanized for biodistribution assays (N = 6) or used for time-activity characterization of aorta signal (N = 3). For the atherosclerosis model, remaining rabbits (N = 36) were placed on Western diet (initial 8 weeks on regular chow enriched with 0.3% cholesterol and 4.7% coconut oil, and the remaining time period on 0.15% enriched cholesterol diet from Research diets, Inc. Brunswick, NJ). In order to induce atherosclerotic lesions, these rabbits were subjected to two endothelial denudations of the aorta through the right and left femoral artery at 2 and 6 weeks after diet initiation, respectively, as described previously^27^. Briefly, femoral artery angioplasty was performed under fluoroscopic guidance using a 4F-Fogarty embolectomy catheter. Once catheter is inserted, catheter is positioned in the thoracic descending aorta region. The balloon was then inflated to 2 atm. Catheter is then pulled back over the entire length of the aorta down to the iliac bifurcation, and the pullback procedure is repeated for two additional times. Catheter is then removed, and femoral artery is ligated. Rabbits with atherosclerosis were imaged 4 months after diet initiation (N = 36). Among these animals some were euthanized for ex vivo biodistribution analyses (N = 9), used for time-activity characterization of aorta signal (N = 3) or near infra-red imaging (N = 27). The remaining atherosclerotic rabbits were fed a Western diet for an additional 3 months (N = 9) to advance atherosclerosis. At the end of the 3-month period, these rabbits were imaged and euthanized.

### Myocardial infarction surgery in mice

Myocardial infarction in mice was induced by permanent ligation of the left anterior descending (LAD) coronary artery of female C57Bl/6 mice (N = 18). Briefly, animals were anesthetized with xylazine (10 mg/kg) and ketamine (100 mg/kg) and intubated using an endotracheal intubation kit from Braintree Scientific (Braintree, MA). Left-sided thoracotomy and pericardial incision were performed. A 7-0 Silk suture was used to occlude the LAD. Incisions were closed with a 5-0 Silk suture. Infarcted animals were treated with 0.1 mg/kg of buprenorphine every 12 h and used 3 days after the surgery.

### [^64^Cu]Cu-DOTATATE biodistribution, pharmacokinetics and blood separation analysis in mice

For biodistribution, pharmacokinetics and blood separation analysis in mice we used [^64^Cu]Cu-labeled DOTATATE due to this isotope’s longer physical half-life compared to [^68^Ga]Ga (12.7 vs. 1.1 h), which allowed us to perform extensive ex vivo validation assays. Wild-type and atherosclerotic animals were injected with approximately 1.85 MBq of [^64^Cu]Cu-DOTATATE and euthanized at either 1, 5, 15, 30, 60 or 120 min after injection (Supplementary Fig. S1A) to evaluate the tracer’s biodistribution. After euthanasia, an insulin syringe was used to collect blood samples from the right ventricle. Blood samples were used for the establishment of pharmacokinetics profile. Mice were then perfused with 20 mL of phosphate-buffered saline (PBS) and tissues of interest (heart, aorta, bone marrow, spleen, liver, stomach, bladder, kidneys, intestines, lungs, adrenal gland, muscles and pancreas) were harvested. Tissues were blotted for gamma counting on a Wizard2 2480 automatic gamma counter (Perkin Elmer, Waltham, MA). Values were corrected for decay and normalized to tissue weight to express radioactivity concentration as percentage injected dose per gram (%ID/g). Blood half-life was calculated by measuring blood radioactivity over time for 120 min and data were fitted using a two-phase decay non-linear regression using GraphPad Prism v8.4.3 (Supplementary Table S1). To investigate the tracer’s distribution within different blood compartments, after gamma counting, 100 μL of blood were spun down at 2000 g for 15 min at 4 °C. Plasma (supernatant) and cells (pellet) were gamma counted and expressed as percentage of total activity. The remainder of the blood was separated using Lymphoprep density gradient medium (Nycomed Pharma, Zurich, Switzerland) following manufacturer’s instructions. Percentage of activity in mononuclear and polynuclear cells was calculated.

### In vivo PET/CT imaging in mice

For the PET/CT experiments, mice were fasted for 12 h before radiotracer injection and anesthetized with xylazine (10 mg/kg) and ketamine (100 mg/kg) through intraperitoneal injection prior to radiotracer administration. Subsequently they were injected via the lateral tail vein with approximately 22.2 MBq of [^18^F]F-FDG or 5.55 MBq of [^64^Cu]Cu-DOTATATE. Tracers were allowed to circulate for 60 min. Immediately before imaging, mice were placed under 1% isoflurane (BaxterHealthcare, Deerfield, IL), and subsequently imaged on a high resolution (700 µm) Mediso nanoScan PET/CT scanner (Mediso, Budapest, Hungary). CT scan was acquired at 50 kVp and 300 ms exposure per projection. eXIA160 (Binitio, Ontario, Canada) was used as a contrast agent to improve imaging of the vasculature by intravenous administration of 100 μl per mouse 5 min prior to CT acquisition^51^. PET acquisition time was 40 min. Reconstruction was performed using TeraTomo 3D reconstruction engine, for 8 iterations and 6 subsets per iteration for both tracers. The voxel size was isotropic at 0.3 mm. Immediately after the PET/CT scan, animals were euthanized for ex vivo assays.

### Flow cytometry

After PET imaging, mouse hearts and aortas were harvested and collected in PBS-filled tubes. Aortas were minced and digested with an enzymatic digestion solution containing liberase TH (4 U/ml) (Roche, Basel, Switzerland), DNase I (40 U/ml) and hyaluronidase (60 U/ml) in PBS. Heart tissue was minced and digested using an enzymatic digestion solution containing DNase I (60 U/ml), collagenase type I (450 U/ml), collagenase type XI (125 U/ml) and hyaluronidase (60 U/ml) in PBS. Samples were treated with the respective enzymatic solution for 60 min at 37 °C. All enzymes were purchased from Sigma-Aldrich (St. Louis, MO). Samples were then passed through a 70 μm filter, washed and prepared for antibody staining and flow sorting. Cellular fragments and debris were gated out of the analysis by utilizing forward and side angle light scatter signal. Macrophages were identified as Ly6G^−^ (Clone 1A8, PE/Cy7), CD11b^+^ (Clone M1/70, PE), CD11c^−^ (Clone N418, PerCP/Cy5.5) and F4/80^+^ (Alexa Fluor^®^ 647, Clone BM8) from Biolegend (San Diego, CA). The remaining CD11b^+^ cells were identified as Ly6G^lo^, CD11b^hi^, CD11c^hi^. Data were acquired on a FACS Aria flow sorter (BD Biosciences, East Rutherford, NJ) and analyzed using FlowJo v10.0.7 (Tree Star, Ashland, OR). Sorted cells were gamma counted and activity per cell values were calculated.

### Autoradiography

Tissues (heart and aorta) were placed in a film cassette against a phosphorimaging plate (BASMS-2325, Fujifilm) at − 20 °C to determine the regional radioactivity distribution. Exposure time was optimized to dose, tracer, and tissue uptake differences. Tissues from the heart of [^18^F]F-FDG injected animals were exposed for 5 min, [^18^F]F-FDG infused aortas were exposed for 30 min and tissues from animals in the [^64^Cu]Cu-DOTATATE group were exposed for 60 min. The plates were read at a pixel resolution of 25 μm with a Typhoon 7000IP plate reader (GE Healthcare, Pittsburgh, PA). Images were analyzed using ImageJ software v1.52 (Madison, WI).

### [^68^Ga]Ga-DOTATATE biodistribution, pharmacokinetics and blood separation analysis in rabbits

Adopting a translational approach, the rabbit studies were performed with the [^68^Ga]Ga-labeled tracer, which is already being used in the clinic. [^68^Ga]Ga-DOTATATE was purchased from Advanced Accelerator Applications (AAA, Millburn, NJ, USA). To determine its pharmacokinetics, three atherosclerotic rabbits and two healthy controls were injected with approximately 190 MBq of [^68^Ga]Ga-DOTATATE. Venous blood was sampled at 1, 5, 10, 15, 20, 30, 60, 120, 180, 240 and 300 min after injection through the ear vein. Samples were weighed and gamma counted. Blood half-life data were fitted using a two-phase decay non-linear regression using GraphPad Prism v8.4.3 (Supplementary Table S1). To investigate the tracer blood distribution, blood samples were centrifuged at 2000 g for 15 min at 4 °C to determine the radioactivity concentration in the plasma and cellular fractions. To analyze [^68^Ga]Ga-DOTATATE biodistribution, all rabbits were euthanized after the last imaging time-point using an injection of pentobarbital 100 mg/kg and exsanguination of the portal vein. Rabbits were extensively perfused with 1000 mL of saline. Following, tissues (blood, heart, aorta, liver, kidney, skeletal muscle, small and large intestine, fat, stomach, pancreas and bladder) were harvested, weighed and gamma counted.

### In vivo PET/MR imaging in rabbits

In vivo imaging in rabbits was performed on a 3 Tesla Biograph mMR (Siemens, Erlangen, Germany) Positron Emission Tomography/Magnetic Resonance Imaging (PET/MRI) clinical scanner. To establish the optimal imaging time-point for [^68^Ga]Ga-DOTATATE aortic imaging in this animal model, 5 rabbits underwent dynamic PET imaging for 3 h immediately after injection of approximately 190 MBq of the tracer. Rabbits where injected intravenously via the ear vein with approximately 190 MBq of either [^18^F]F- FDG or [^68^Ga]Ga-DOTATATE. Rabbits undergoing [^18^F]F-FDG scans were fasted for 3–4 h before injection to avoid tracer uptake in the bowels, as previously validated^52^. After injection, [^18^F]F-FDG was allowed to circulate for 3 h, as per previously described^28^, while, based on results of dynamic analyses, [^68^Ga]Ga-DOTATATE was left to circulate for 2 h. At the end of the circulation time, rabbits were anesthetized with ketamine (20 mg/kg) and xylazine (5 mg/kg) i.m. Animals were then placed in supine position under 1.5% isoflurane inhalation on the PET/MR scanner. After scout scans, PET imaging was initiated for 30 min for [^18^F]F-FDG and 60 min for [^68^Ga]Ga-DOTATATE. 3D non contrast enhanced time-of-fight (TOF) images were acquired to visualize the abdominal aorta and renal arteries, with the following imaging parameters: repetition time (TR), 23 ms; echo time (TE), 2.8 ms; flip angle, 20 degrees; spatial resolution, 0.35 mm^2^ (interpolated); slice thickness, 1 mm. PET images were reconstructed offline. Attenuation correction of PET images was performed by using a vendor built-in Dixon MR- based attenuation map (MR-AC) with 2 (soft tissue, air). Images were reconstructed using a 3D ordinary Poisson ordered subsets expectation maximization (OP-OSEM) algorithm with point-spread-function (PSF) resolution modeling, using 3 iterations and 21 subsets and filtered with a 4 mm Gaussian filter. Animals were then euthanized, perfused and aortas were collected for gamma counting.

### [^64^Cu]Cu-DOTATATE, [^68^Ga]Ga-DOTATATE and [^18^F]F-FDG PET image analysis

Using OsiriX v5.6 software (OsiriX Foundation, Geneva, Switzerland), regions of interest (ROIs) were drawn to determine organ radioactivity concentration. In mice, for healthy and infarcted myocardium, ROIs were traced manually in the apex of the heart in the axial view. For remote myocardium, analysis was performed in myocardium 180° from the infarcted zone. In the mice aorta, sequential ROIs were drawn in the ascending aorta based on consecutive tomographic (CT) slices beginning from the root up to the brachiocephalic branch, merging point with the aortic arch. Voxel counts were converted to maximum standardized uptake values (SUV_max_). In rabbits, PET images were fused with time-of-flight bright blood MRI angiography and ROIs were manually drawn on the infrarenal abdominal aorta in TOF images. The slice-by-slice maximum standard uptake values (SUV_max_) were averaged across the whole aorta. When prominent image artifacts, such as spill over artefacts into the abdominal aorta from neighbouring tissues, were present, rabbits were excluded from image analysis, as detailed in the Results section. To ensure blinding, investigator involved in the imaging analysis was not aware of the group distribution in the imaging slices.

### Near infrared fluorescence imaging in of rabbit aortas

Ex vivo near infrared fluorescence imaging of rabbit aortas was performed on a Xenogen IVIS Spectrum Preclinical Imaging System (Perkin Elmer, Waltham, MA). Approximately 24 h before euthanasia, animals were injected with Cy5.5-labeled high-density lipoprotein (HDL), which was used to determine macrophage burden. Fluorescence images were obtained with the following excitation and emission band-pass filter parameters: λ excitation = 640 ± 18 nm, λ emission = 720 ± 10 nm. Images were subsequently analyzed by dividing the infrarenal abdominal aorta into 8 ROIs of the same size. Max radiant efficiency [(p/s/cm^2^/sr)/(μW/cm^2^)] for each ROI were recorded as a measure Cy5.5-HDL deposition in the aorta.

### Statistics

All results are presented as mean ± standard error of the mean (SEM) or median and interquartile range (IQR). After running appropriate tests for normality, non-parametric statistical tests were deemed appropriate. Unpaired data were analyzed with non-parametric Mann–Whitney tests. Analysis of longitudinal imaging studies were performed using Wilcoxon matched-pairs signed rank tests. Correlation of [^68^Ga]Ga-DOTATATE maximum standardized uptake value (SUV_max_) and [^18^F]F-FDG SUV_max_ was calculated by computing a nonparametric Spearman coefficient. For all tests, α < 0.05 represents statistical significance. Levels of significance were indicated as follows: *p < 0.05, **p < 0.01, ***p < 0.001, ****p < 0.0001.

## Supplementary Information

Supplementary Information.

## Data Availability

The datasets generated during and/or analysed during the current study are available from the corresponding author on reasonable request.
