# Supplementary material for: Systematically evaluating DOTATATE and FDG as PET immuno-imaging tracers of cardiovascular inflammation
Source: Sci Rep. 2022 Apr 13;12:6185. doi: 10.1038/s41598-022-09590-2 (PMC9007951; doi:10.1038/s41598-022-09590-2)
Supplement: Supplementary file 1 — Supplementary Information. [file 41598_2022_9590_MOESM1_ESM.docx]

Systematically evaluating DOTATATE and FDG as PET immuno-imaging tracers of cardiovascular inflammation

Yohana C. Toner^1,2,3‡^, Adam A. Ghotbi^1,2,4‡^, Sonum Naidu^1,2^, Ken Sakurai^1,2^, Mandy M.T. van Leent^1,2^, Stefan Jordan^5,6^, Farideh Ordikhani^5^, Letizia Amadori^7,8^, Alexandros Marios Sofias^1,2,9^, Elizabeth L. Fisher^1,2^, Alexander Maier^1,2,10^, Nathaniel Sullivan^1,2^, Jazz Munitz^1,2^, Max L. Senders^1,2,11^, Christian Mason^12^, Thomas Reiner^12,13,14^, Georgios Soultanidis^1,2^, Jason M. Tarkin^15^, James H.F. Rudd^15^, Chiara Giannarelli^7,8,16^, Jordi Ochando^5,17^, Carlos Pérez-Medina^1,2,18^, Andreas Kjaer^4^, Willem J.M. Mulder^1,2,3,19^, Zahi A. Fayad^1,2^, Claudia Calcagno^1,2*^

^1^BioMedical Engineering and Imaging Institute, Icahn School of Medicine at Mount Sinai, New York, NY, USA ^2^Diagnostic, Molecular and Interventional Radiology, Icahn School of Medicine at Mount Sinai, New York, NY, USA ^3^Department of Internal Medicine and Radboud Center for Infectious Diseases, Radboud University Medical Center, Nijmegen, the Netherlands ^4^Department of Clinical Physiology, Nuclear Medicine & PET and Cluster for Molecular Imaging, Rigshospitalet and University of Copenhagen, Copenhagen, Denmark ^5^Department of Oncological Sciences, Icahn School of Medicine at Mount Sinai, New York, NY, USA ^6^Charité *–* Universitätsmedizin Berlin, corporate member of Freie Universität Berlin and Humboldt-Universität zu Berlin, Institute of Microbiology, Infectious Diseases and Immunology, Berlin, Germany ^7^Department of Genetics and Genomic Sciences Icahn School of Medicine at Mount Sinai, New York, NY, USA ^8^New York University Cardiovascular Research Center, Department of Medicine, Leon H. Charney Division of Cardiology, New York University Grossman School of Medicine, New York University Langone Health, New York, NY, USA ^9^Department of Circulation and Medical Imaging, Faculty of Medicine and Health Sciences, Norwegian University of Science and Technology (NTNU), Trondheim, Norway ^10^Heart Center Freiburg University, Department of Cardiology and Angiology I, Faculty of Medicine, University of Freiburg, Freiburg, Germany ^11^Department of Medical Biochemistry, Academic Medical Center, Amsterdam, Netherlands ^12^Department of Radiology, Memorial Sloan-Kettering Cancer Center, New York, NY, USA ^13^Department of Radiology and Chemical Biology Program, Memorial Sloan Kettering Cancer Center, New York, NY, USA ^14^Department of Radiology, Weill Cornell Medical College, New York, NY, USA ^15^Division of Cardiovascular Medicine, University of Cambridge, Cambridge, UK ^16^Cardiovascular Research Center, Department of Medicine, Icahn School of Medicine at Mount Sinai, New York, NY, USA ^17^Transplant Immunology Unit, National Center of Microbiology, Instituto de Salud Carlos III, Madrid, Spain ^18^Centro Nacional de Investigaciones Cardiovasculares (CNIC), Madrid, Spain ^19^Laboratory of Chemical Biology, Department of Biochemical Engineering, Eindhoven University of Technology, Eindhoven, The Netherlands.

^‡^ The authors contributed equally to this work

*Corresponding author: Claudia Calcagno. 1470 Madison Ave, NY 10029. PO Box: 1234. Fax: +1 (240) 368-8096. Phone: +1 (212) 824-8464. claudia.calcagno@mssm.edu

**Supplemental material**

**SUPPLEMENTAL FIGURES**

**Supplementary Figure S1.** Flowchart of the experimental design in mice. A) C57Bl/6 and *Apoe*^-/-^ mice at 16-20 weeks of age were injected with [^64^Cu]Cu-DOTATATE and euthanized at different time-points. Animals were perfused and organs of interest were gamma-counted. Blood was processed for plasma, mononuclear and polynuclear cell separation. Results were plotted as %ID/g. B) Healthy and MI (3 days after infarct) C57Bl/6 mice, and *Apoe*^-/-^ mice were injected with either [^18^F]F-FDG or [^64^Cu]Cu-DOTATATE. Tracer was allowed to circulate for 60 minutes. Animals were injected with eXIA™160 contrast agent (Binitio Biomedical Inc, Ontario, Canada). PET/CT acquisition was performed for 40 minutes. Mice were then sacrificed and perfused. Aorta and heart, the organs of interest, were collected and flow sorted. After sorting, cells were gamma-counted and activity per cell ratio was calculated. ID: injected dose.

**Supplementary Figure S2** Flowchart of the experimental design in rabbits. A) Healthy and atherosclerotic (athero_4mo_) New Zealand White rabbits were injected with [^68^Ga]Ga-DOTATATE and pharmacokinetics profile was determined by blood sampling at different time-points. Blood was processed for plasma and cell separation. At 180 minutes, animals were sacrificed and perfused. Organs of interest were gamma-counted. Results were plotted as %ID/g. B) Healthy and atherosclerotic (athero_4mo_) New Zealand White rabbits were injected with either [^68^Ga]Ga-DOTATATE or [^18^F]F-FDG. Tracer was allowed to circulate for 120 ([^68^Ga]Ga-DOTATATE) and 180 minutes ([^18^F]F-FDG), after which, rabbits were imaged with PET/MR for 60 or 30 minutes, respectively. Control animals and athero_4mo_ were then sacrificed and perfused. Organs of interest were gamma counted and aortas were imaged with near infrared. A group of 9 atherosclerotic animals (athero_7mo_) was kept and fed with Western Diet for additional 3 months, after which animals were imaged again with [^68^Ga]Ga-DOTATATE and [^18^F]F-FDG, sacrificed and perfused for aorta harvesting. Aortas were imaged with near infrared. Athero surgery: balloon denudation surgery; ID: injected dose; WD: Western Diet.

**Supplementary Figure S3.** [^64^Cu]Cu-DOTATATE uptake in mice and autoradiography of [^64^Cu]Cu-DOTATATE and [^18^F]F-FDG in cardiovascular disease animal models. A) Heatmap visualization of [^64^Cu]Cu-DOTATATE distribution at different timepoints in healthy C57Bl/6 (left) and atherosclerotic *Apoe*^-/-^ (right) mice (N=5 per group). Data was obtained by *ex vivo* gamma counting of blotted organs. B) Representative autoradiography images showing radioactivity distribution in the myocardium of MI mice injected with [^18^F]F-FDG or [^64^Cu]Cu-DOTATATE. Regions of interest (ROI) represented in red (infarcted myocardium) and black (non-infarcted remote myocardium). Quantification of signal was plotted as ratio between infarcted and remote myocardium. (N=4 per group) C) Representative plot of gating strategy for flow sorting of heart (top) and aorta (bottom) tissues D) Representative autoradiography images showing radioactivity distribution in the aorta of C57Bl/6 mice and *Apoe*^-/-^ mice injected with [^18^F]F-FDG or [^64^Cu]Cu-DOTATATE. Regions of interest (ROI) represented in red (plaque) and black (no plaque). Quantification of signal was plotted as ratio between plaque and no plaque signal. (N=4 per group). cps: counts per second; DOTATATE: [^64^Cu]Cu-DOTATATE; FDG: [^18^F]F-FDG; ID: injected dose. Data are presented as median (interquartile range).

**Supplementary Figure S4.** Analysis of [^68^Ga]Ga-DOTATATE biodistribution in rabbits and [^18^F]F-FDG and [^68^Ga]Ga-DOTATATE aortic uptake comparison. A) [^68^Ga]Ga-DOTATATE radioactivity distribution in blood fractions of control (left) and athero_4mo_ (right) rabbits as determined by gamma counting. Graphs show the percentage of activity associated with cells or plasma over time. Controls N=2 and athero_4mo_ N=3 B) Heatmap visualization of [^68^Ga]Ga-DOTATATE distribution at different timepoints in healthy (left) and athero_4mo_ rabbits (right) Controls N=6 and athero_4mo_ N=9. Data was obtained by *ex vivo* gamma counting of blotted organs. C) [^68^Ga]Ga-DOTATATE SUV_max_ of abdominal aorta in control and athero_4mo_ animals over the first 3 hours after injection, acquired by dynamic PET/MR imaging. N=3 per group. Data are presented as mean ± standard error of the mean. D) Correlation between [^18^F]F-FDG and [^64^Cu]Cu-DOTATATE aortic uptake in control, athero_4mo_ and athero_7mo_ animals. N=57. DOTATATE: [^64^Cu]Cu-DOTATATE; FDG: [^18^F]F-FDG; ID: injected dose; SUV_max_: maximum standardized uptake value.

**SUPPLEMENTARY TABLE S1**. Blood circulation half-lives of DOTATATE-based radiotracers in mice and rabbits.

|  | Mice | | Rabbits | |
| --- | --- | --- | --- | --- |
|  | C57Bl/6 | *Apoe^-/-^* | Control | Athero_4mo_ |
| %fast | 79.14 | 11.27 | 39.7 | 53.17 |
| t_1/2_ fast (minutes) | 1.939 | 0.557 | 3.419 | 19.17 |
| %slow | 20.86 | 88.73 | 60.3 | 46.83 |
| t_1/2_ slow (minutes) | 15.81 | 7.627 | 32.86 | 92.82 |
| Weighed t_1/2_ (minutes) | 4.83 | 6.83 | 21.17 | 53.66 |

**SUPPLEMENTARY TABLE S2**. Tissue radioactivity distribution of [^64^Cu]Cu-DOTATATE in female C57BL/6 mice in %ID/g.

| WT | 1 min | 5 min | 15 min | 30 min | 60 min | 120 min |
| --- | --- | --- | --- | --- | --- | --- |
| Bone marrow | 6.5 (5.4-7.1) | 4.3 (3.6-5.6) | 3.5 (3-5.6) | 1.9 (1.7-2.9) | 2.1 (1.6-2.5) | 2.5 (2.1-4.1) |
| Liver | 10 (8.5-11.1) | 7.1 (6.1-7.7) | 6.5 (6.1-7) | 6.4 (6-6.4) | 6.9 (6.7-7.8) | 5.4 (4.8-6.4) |
| Spleen | 3.7 (3.2-4.2) | 1.5 (1.2-2) | 0.9 (0.8-0.9) | 0.7 (0.7-0.8) | 0.6 (0.6-0.8) | 0.5 (0.5-0.7) |
| Pancreas | 8.7 (7.5-9.3) | 5.3 (4.7-6.3) | 6.1 (5.7-6.4) | 5.8 (5.2-6.2) | 4.8 (4.5-5) | 2.6 (2.5-3.4) |
| Stomach | 2.3 (2.2-3.1) | 2.4 (1.7-3.2) | 5 (3.5-7) | 7.8 (3.6-8.7) | 8 (4.8-10.8) | 3.2 (2.7-3.6) |
| Fat | 1.1 (0.5-1.5) | 1.3 (1.1-1.5) | 0.8 (0.6-0.9) | 0.5 (0.4-0.8) | 0.3 (0.2-0.4) | 0.2 (0.2-0.5) |
| Bladder | 4.6 (2.5-24) | 36.4 (8.2-78.9) | 7.9 (6.1-34.9) | 6.8 (4-12.6) | 3.3 (1.9-6.6) | 10.4 (1.5-15.2) |
| Kidneys | 26.8 (19.1-33.4) | 20.5 (18.7-24.2) | 11.5 (11.3-13.6) | 8.2 (7.3-10.9) | 6.1 (3-6.7) | 3.7 (3.3-4.6) |
| Adrenal gland | 3.3 (2.9-3.5) | 2.2 (2.1-2.5) | 1.7 (1.4-2.2) | 1.6 (1.5-1.7) | 1.6 (1.5-1.8) | 0.6 (0.6-1.7) |
| Lungs | 1.8 (1.2-2.1) | 3.2 (2.7-4) | 0.9 (0.7-2) | 1.3 (1.1-2.2) | 1.1 (0.8-1.1) | 1.8 (1.2-2.6) |
| Tail | 3.5 (2-4.9) | 2.7 (1.6-4) | 3.6 (2.9-3.7) | 1.8 (1.6-2.9) | 1.6 (0.6-1.7) | 0.6 (0.5-0.7) |
| Muscle | 2 (1.7-2.5) | 1.6 (1.5-2.1) | 0.9 (0.7-0.9) | 0.5 (0.3-0.6) | 0.3 (0.3-0.6) | 0.3 (0.2-0.7) |
| Large intestine | 2.7 (2.3-3.7) | 1.6 (1.4-1.9) | 1.9 (1.7-2.2) | 1.7 (1.3-2.1) | 3 (2.5-3.3) | 3.1 (1.8-3.5) |
| Small intestine | 3 (2.3-3.6) | 2 (1.6-2.1) | 1.7 (1.3-2.4) | 2.6 (1.6-4.4) | 2.4 (0.9-3.7) | 3.3 (3.1-3.8) |
| Lymph node | 3 (2.8-3.8) | 4.7 (4.5-5.5) | 3.2 (2.8-3.9) | 2.2 (1.5-4.7) | 1.3 (1.2-1.7) | 0.6 (0.4-1.8) |
| Heart | 3.8 (3.6-4.9) | 2.1 (1.9-2.5) | 1.1 (0.9-1.2) | 0.9 (0.8-1) | 0.8 (0.4-0.9) | 0.7 (0.7-0.8) |
| Thoracic aorta | 3.2 (2.9-4.6) | 3.1 (2.9-3.5) | 1.6 (1.4-2.4) | 1.3 (1.2-1.4) | 1.2 (1.2-1.4) | 1 (0.7-1.1) |
| Abdominal aorta | 5.1 (4.1-5.3) | 3.8 (3-4) | 2.4 (2.4-3.2) | 1.7 (1.3-2.1) | 1.3 (1.1-1.8) | 0.7 (0.3-1.9) |
| Aortic arch | 2.8 (2.7-4.5) | 2.2 (1.8-2.9) | 1.3 (1.2-2.1) | 1.1 (1-1.2) | 1.1 (1-1.1) | 1.2 (1.2-1.6) |

(N=3-7 per time-point). Data are presented as median (interquartile range).

**SUPPLEMENTARY TABLE S3**. Tissue radioactivity distribution of [^64^Cu]Cu-DOTATATE in female *Apoe^-/-^* mice 12 weeks on Western Diet represented in %ID/g.

| *Apoe-/-* | 1 min | 5 min | 15 min | 30 min | 60 min | 120 min |
| --- | --- | --- | --- | --- | --- | --- |
| Bone marrow | 3.1 (2.2-3.5) | 2.3 (1-4) | 2.6 (2.1-3.2) | 1.8 (1.5-3.1) | 1.9 (1.2-2.4) | 2.7 (2-3) |
| Liver | 4.4 (4.1-4.9) | 4.9 (4.1-6.5) | 8.6 (6.4-12.6) | 6.9 (6.6-7.4) | 6.9 (5.9-8.7) | 6.3 (5.6-7.5) |
| Spleen | 2 (1.6-2.5) | 1.1 (0.8-1.3) | 1.2 (0.8-1.6) | 1.1 (0.8-1.2) | 0.8 (0.6-1.2) | 1.2 (1.1-1.3) |
| Pancreas | 4.7 (3.9-6) | 4.1 (3-4.4) | 3.5 (2.9-4.2) | 3.4 (3.1-3.8) | 2.7 (2.3-3.5) | 2.6 (1.9-3.2) |
| Stomach | 2.1 (1.6-2.6) | 2.4 (1.8-3.2) | 3.3 (3.1-4.7) | 5.4 (4.1-6.7) | 5.4 (4.1-9.4) | 5 (4-6.8) |
| Fat | 0.3 (0.3-0.3) | 0.4 (0.3-0.7) | 0.4 (0.3-0.5) | 0.3 (0.2-0.6) | 0.2 (0.2-0.7) | 0.2 (0.2-0.4) |
| Bladder | 1.7 (1.6-2.1) | 98.7 (69.1-165) | 38.9 (16-63.1) | 2.9 (1.8-3.6) | 4.7 (2.5-17.5) | 2.4 (1.4-10.2) |
| Kidneys | 15.6 (15.1-17.5) | 22.9 (18.7-48.4) | 13.2 (11.9-16) | 8.8 (7.3-10.5) | 11.2 (6-11.6) | 7.1 (7-8.1) |
| Adrenal gland | 1.4 (1.2-1.5) | 1.2 (1-1.5) | 1.4 (1.2-1.7) | 1.5 (1.4-1.9) | 1.4 (1.1-1.6) | 1.1 (0.7-1.5) |
| Lungs | 2.1 (2.1-3.3) | 2.6 (2.2-3) | 1.9 (0.9-2.7) | 1.6 (1.3-2.5) | 2.5 (1.6-3.1) | 2.8 (2.2-3.5) |
| Tail | 1.8 (1-2.7) | 2.5 (1.7-4.5) | 2.5 (1.6-4.7) | 3.1 (1.6-3.3) | 2 (1.5-5.9) | 2.7 (1.3-3.4) |
| Muscle | 1.2 (1.1-1.5) | 1.4 (1.3-2.2) | 0.6 (0.5-0.9) | 0.7 (0.5-1.4) | 0.4 (0.3-0.6) | 0.3 (0.3-0.7) |
| Large intestine | 1.3 (1-1.5) | 1.3 (1.1-1.6) | 1.8 (1.1-2.3) | 2.8 (1.4-3.9) | 2.2 (1.1-3.1) | 3.8 (3.6-6.5) |
| Small intestine | 1.4 (0.7-3) | 1.2 (1-1.7) | 1.8 (1-2) | 3.1 (1.8-5.6) | 7.1 (4.7-7.8) | 7.2 (4.4-9.5) |
| Lymph node | 2.6 (1.8-3.8) | 3.2 (2.5-4) | 3 (2.2-4.1) | 2.4 (1.6-4.6) | 2.1 (1.3-2.7) | 1.8 (1.4-2.6) |
| Heart | 2.3 (2.1-2.8) | 1.4 (0.7-1.6) | 1.1 (0.8-1.3) | 0.9 (0.8-1.2) | 1.3 (1.1-1.5) | 1.4 (1.2-1.4) |
| Thoracic aorta | 2.9 (2.5-3.2) | 2.1 (1.9-2.4) | 2 (1.8-2.3) | 1.8 (1.6-2.3) | 2.1 (1.6-2.2) | 2.1 (2-2.4) |
| Abdominal aorta | 3.1 (2.9-3.6) | 3.2 (2.6-4.6) | 2.1 (1.7-3) | 2.5 (1.9-3.6) | 2.2 (1.7-2.3) | 2.1 (1.9-2.5) |
| Aortic arch | 2.9 (2.4-3) | 1.9 (1.9-1.9) | 1.7 (1.6-2) | 1.7 (1.5-2.1) | 1.8 (1.3-2) | 1.9 (1.9-1.9) |

(N=3-8 per time-point). Data are presented as median (interquartile range).

**SUPPLEMENTARY TABLE S4.** Number of cells analyzed by flow cytometry.

| Tissue | Cells | Mouse 1 | Mouse 2 | Mouse 3 | Mouse 4 |
| --- | --- | --- | --- | --- | --- |
| Infarcted myocardium C57Bl/6 (FDG) | Macrophages | 13644 | 3871 | 3591 | 1400 |
|  | Other CD11b positive | 55751 | 12479 | 7031 | 18511 |
|  | CD11b negative | 403562 | 182839 | 264546 | 145387 |
|  | Total | 472957 | 199189 | 275168 | 165298 |
| Infarcted myocardium C57Bl/6 (DOTA) | Macrophages | 22882 | 5168 | 7279 | 6314 |
|  | Other CD11b positive | 65733 | 39530 | 35666 | 30890 |
|  | CD11b negative | 602928 | 225498 | 466014 | 255403 |
|  | Total | 691543 | 270196 | 508959 | 292607 |
| Aorta *Apoe^-/-^* (FDG) | Macrophages | 6414 | 3288 | 2275 | 1429 |
|  | Other CD11b positive | 24293 | 15900 | 22842 | 9988 |
|  | CD11b negative | 435993 | 855828 | 405410 | 191276 |
|  | Total | 466700 | 875016 | 430527 | 202693 |
| Aorta *Apoe^-/-^* (DOTA) | Macrophages | 4281 | 3370 | 7270 | 6917 |
|  | Other CD11b positive | 22812 | 21362 | 44521 | 40724 |
|  | CD11b negative | 513480 | 179121 | 392296 | 321097 |
|  | Total | 540573 | 203853 | 444087 | 368738 |

**SUPPLEMENTARY TABLE S5**. Tissue radioactivity distribution of [^68^Ga]Ga-DOTATATE in male control rabbits and athero_4mo_ in %ID/g.

|  | Controls  Median (interquartile range) | Athero_4mo_  Median (interquartile range) |
| --- | --- | --- |
| Bone marrow | 0.003 (0.002-0.009) | 0.006 (0.003-0.012) |
| Liver | 0.009 (0.005-0.013) | 0.006 (0.004-0.007) |
| Spleen | 0.015 (0.007-0.035) | 0.012 (0.011-0.013) |
| Pancreas | 0.005 (0.004-0.009) | 0.009 (0.007-0.011) |
| Stomach | 0.036 (0.022-0.071) | 0.053 (0.041-0.079) |
| Fat | 0 (0-0.001) | 0.002 (0.002-0.005) |
| Bladder | 0.053 (0.014-0.1) | 0.075 (0.038-0.155) |
| Kidneys | 0.128 (0.108-0.148) | 0.157 (0.141-0.263) |
| Adrenal gland | 0.003 (0.003-0.005) | 0.012 (0.009-0.017) |
| Lungs | 0.005 (0.003-0.012) | 0.015 (0.012-0.02) |
| Muscle | 0.001 (0.001-0.002) | 0.003 (0.002-0.006) |
| Large intestine | 0.005 (0.004-0.012) | 0.01 (0.007-0.015) |
| Small intestine | 0.025 (0.016-0.033) | 0.021 (0.016-0.029) |
| Lymph node | 0.005 (0.005-0.007) | 0.013 (0.01-0.019) |
| Heart | 0.003 (0.003-0.005) | 0.006 (0.005-0.008) |
| Thoracic aorta | 0.004 (0.002-0.006) | 0.015 (0.011-0.018) |
| Abdominal aorta | 0.006 (0.003-0.02) | 0.017 (0.014-0.022) |

(N=6-9 per group). Data are presented as median (interquartile range).
